# Profiling chronic diseases and hospitalizations in older home care recipients: a nationwide cohort study in Sweden

**DOI:** 10.1186/s12877-024-04796-7

**Published:** 2024-04-03

**Authors:** Katharina Schmidt-Mende, Cecilia Arvinge, Giovanni Cioffi, Lars Lennart Gustafsson, Karin Modig, Anna Carina Meyer

**Affiliations:** 1 Academic Primary Health Care Centre, Region Stockholm, Stockholm, Sweden; 2 Department of Neurobiology and Care Sciences and Society, Division of Family Medicine and Primary Care, Karolinska Institutet, Huddinge, Sweden; 3 Torsvik Primary Health Care Centre, Region Stockholm, Lidingö, Sweden; 4 Institute of Environmental Medicine, Unit of Epidemiology, Karolinska Institutet, Stockholm, Sweden; 5 Department of Laboratory Medicine, Division of Clinical Pharmacology, Karolinska Institutet, Huddinge, Sweden

**Keywords:** Aged, Multimorbidity, Primary health care, Home care, Hospitalization

## Abstract

**Background:**

Older adults with home care (HC) often have complex disease patterns and use healthcare extensively. Increased understanding is necessary to tailor their care. To our knowledge, this is the first study to describe patterns of morbidity and hospitalizations among community-dwelling older HC recipients nationwide and in subgroups defined by age, sex, and amount of HC, and to compare patterns to community-dwelling older adults without HC.

**Methods:**

Nationwide register-based cohort study in community-dwelling adults aged 70 and older receiving publicly funded HC in Sweden on January 1st 2019 and an age-and-sex matched comparison group (“non-HC recipients”). Using register data from inpatient and specialized outpatient care, we assessed the prevalence of sixty chronic diseases, frailty, multimorbidity and hospitalizations, calculated incidence rates and explored reasons for hospitalizations during two years of follow-up.

**Results:**

We identified 138,113 HC recipients (mean age 85, 66% women, 57% ≥5 chronic diseases). The most prevalent diseases were hypertension (55%) and eye conditions (48%). Compared to non-HC recipients, HC recipients had a higher prevalence of almost all diseases, with an overrepresentation of neurological (26.1 vs. 9.5%) disease and dementia (9.3 vs. 1.5%). 61% of HC recipients were hospitalized at least once during two years, which was 1.6 times as often as non-HC recipients. One third of HC recipients´ hospitalizations (37.4%) were due to injuries, infections, and heart failure. Hospitalizations for chronic obstructive pulmonary disease, confusion, infections, and breathing difficulties were 3–5 times higher among HC recipients compared to non-HC recipients.

**Conclusion:**

Compared to non-HC recipients, HC recipients more often live alone, have higher degrees of frailty, suffer from more chronic diseases, especially neurological disease, and are hospitalized almost twice as often. The results provide a thorough description of HC recipients, which might be useful for targeted healthcare interventions including closer collaboration between primary care, neurologists, and rehabilitation.

**Supplementary Information:**

The online version contains supplementary material available at 10.1186/s12877-024-04796-7.

## Background

Most older adults have multiple long-term conditions [1], which is linked to various degrees of reduced functional autonomy [2]. In 2019, every other European aged 65 and older reported at least one difficulty in activities of daily living [3]. In order to allow older adults with functional impairment to live at home, Sweden offers publicly funded *home care (HC)* to older adults in need (Box 1) [4]. Since HC access is needs-based [5], recipients presumably present with even more complex health care needs than the average older person [6] with higher risks for hospitalizations and death [7, 8].

**Box 1:** Organization of social care in Sweden

Sweden has a tax funded social care system that is regulated through the Social Services Act (S*ocialtjänstlagen*) [9]. The principle of social care is to provide care based on needs to every citizen. Social care is provided either in people’s homes (*home care*) or at nursing homes. Since the 1990s, there has been a dramatic shift in the care of frail older adults as the number of nursing home beds has decreased [10]. Today, most older citizens are cared for in their homes as a result of policies aiming to promote “*Ageing in place*” [11].
Home care is provided by Swedish municipalities and includes both instrumental help, e.g., with housekeeping or managing finances, as well as personal care, e.g., with dressing, meal assistance, or with hygiene. Healthcare, on the other hand, is provided by Swedish regions. There is limited collaboration between these authorities. Case managers at municipalities assessing the need for home care have no access to healthcare information, and healthcare professionals are not aware if a patient receives home care.

In Sweden and elsewhere, HC and healthcare are provided by different authorities (Box 1) which challenges the provision of integrated quality care that matches older adults’ needs and prevents unnecessary care utilization [12]. Primary care (PC) plays an important role in ensuring coordinated care for community-dwelling older adults [13, 14] and in preventing emergency department visits and hospitalizations. However, data are scanty on morbidity patterns among Swedish HC recipients and on reasons for which they may seek hospital care, which makes it difficult to plan for and tailor HC recipients’ care. A Finnish study found that several conditions were common among newly registered HC recipients, particularly Alzheimer’s dementia (20%), heart failure (18%), and stroke (5%). Of these HC recipients, 43% were hospitalized during one year [15].

With an ageing population and an ageing-in-place policy adopted in many countries, it is important to understand the health status and conditions of this growing population group. We are, however, not aware of any nationwide study presenting morbidity patterns and healthcare use in the entire HC population while considering potential heterogeneity. In this study, we provide a thorough description of present sociodemographic characteristics, disease patterns, and hospitalizations among all older HC recipients in Sweden and among subgroups defined by age, sex, and amount of provided HC. Moreover, in order to get a perception of the disease burden of the HC population, we additionally, provide the corresponding information for the general population of the same age and sex but without HC as a comparison. Increased knowledge in this area is not only important to inform PC practitioners and HC managers but also to develop targeted interventions and training of HC staff.

## Methods

### Study population and data sources

This nationwide Swedish cohort study is based on an extensive database created through linkage of several population registers (Table S1). This database contains health, socioeconomic, and demographic information for the entire population of older adults registered in Sweden followed until the end of 2020. Notable data sources include the National Patient Register [16], which records all inpatient and specialized outpatient care contacts within Sweden classified according to International Classification of Diseases (ICD) codes, and the Social Service Register [17], which collects data on publicly funded long-term care for older adults in Sweden.

We identify all individuals aged 70 + who had a granted formal HC claim on January 1, 2019. It is possible, or even likely, that both people with (formal) HC and those without (formal) HC may have received informal care from family. This is, however, not possible to study since data on this is not available. The provision of security alarm without further HC visits is not considered as HC. To compare the disease burden among HC recipients to other older adults, we identify a comparison group with identical age- and sex-distribution in the general population but without HC. Individuals migrating internationally since 2014 were excluded. The study population is followed until the end-of-follow-up on December 31, 2020 or until admission to nursing homes, emigration, or death, whichever comes first.

## Variables

HC is defined as all publicly funded care from social services but does not include medical care provided by general practitioners or other medical staff in the older person’s home. In our study, receiving HC is defined as having a granted claim with > 0 monthly hours of HC registered in the Social Service Register. HC recipients are categorized into groups with low (< 10 h per month), medium (10-39 h per month), and high (40 + per month) utilization of HC, roughly representing tertiles of the distribution of HC hours in the population. Mortality is measured through death records in the cause of death registry.

To measure morbidity, our study includes 60 clinically relevant long-term conditions, based on an algorithm developed by an expert committee of physicians, geriatricians, and epidemiologists frequently used in international research [18]. We considered all ICD-10 codes registered in the National Patient Register (inpatient and specialized outpatient care) during 2014-18, including main diagnoses and up to 29 secondary diagnoses, to assess disease prevalence. Based on these data, we also calculated frailty scores according to an established algorithm to capture frailty using routinely collected health records [19].

All hospitalizations during two years of follow-up are identified in the National Patient Register. Outpatient visits are not considered when assessing the number of, or reasons for, hospitalizations. The reason for hospitalization is defined as the first recorded main diagnosis registered during the hospital stay.

### Statistical analyses

Statistical significance of differences in disease prevalence between HC groups was tested using χ2-tests (prevalences) and Poisson regression (incidence rates). For each HC recipient, we randomly selected a person of the same age and sex without HC in the community from the Swedish population as the comparison group (in the following called *non-HC recipients*). Ages 100 + were grouped into one category due to the small number. To calculate the number of hospitalizations, we counted all hospital admissions occurring at least one day after a previous discharge (i.e., we considered direct transfers from one clinic to another as a single admission). Incidence rates were calculated as number of hospitalizations divided by person-time at risk during 2019–2020. To compare hospitalization frequency among HC and non-HC recipients, we calculated incidence rate ratios (IRR) as incidence rates among HC recipients divided by incidence rates among non-HC recipients. To explore whether the COVID-19 pandemic affected hospitalization patterns, we restricted the follow-up period to the year 2019 in the sensitivity analyses. We further conducted sensitivity analyses including all main diagnoses recorded during hospitalizations, rather than the first diagnosis made upon admission. Data analyses were conducted with Stata (StataCorp LLC, College Station, TX) and R version 4.2.3 (R Foundation for Statistical Computing, Vienna, Austria).

### Ethical approval

This study was approved by the Regional Ethics Committee in Stockholm (permit numbers Dnr 2011/136 − 31/5 and Dnr 2015/1917-32). The Committee waived the need for patient consent.

## Results

The study population consists of 136,113 HC recipients with a mean age of 85 years of which 65.9% were women (Table 1), corresponding to 8.8% of the Swedish population aged 70 and older.

**Table 1:** Demography, marital status and morbidity among home care recipients and an age-and-sex matched cohort without home care (*N* = 272,226), Sweden 2019-20

	No home care	Home care (Total)	Home care		
			Low amount	Medium amount	High amount
	*N* = 136,113	*N* = 136,113	*N* = 43,538	*N* = 45,705	*N* = 46,870
**Men (%)**	34.1	34.1	35.5	33.6	33.2
**Age (%)**					
70–79	25.5	25.5	27.9	26.4	25.5
80–89	49.1	49.1	51.9	48.8	46.7
90+	24.4	24.4	20.2	24.8	27.8
**Home care**					
Median hours (IQR)	N/A	22 (6;53)	4 (2;6)	21 (15;29)	69 (52;97)
Personal care (%)	N/A	72.7	46.0	84.1	86.4
Instrumental help (%)	N/A	71.6	68.9	71.8	73.9
Safety alarm (%)	16.9	62.1	52.5	66.3	67.0
**Mortality within 1 year (%)**	4.7	13.9	8.9	13.0	19.6
**Mortality within 2 years (%)**	11.1	28.9	19.9	27.4	38.6
**Cohabiting (%)**	49.8	28.3	32.5	26.6	25.8
**Foreign born (%)**	12.1	12.6	12.2	11.3	14.4
**Gilbert frailty score (%)**					
0	80.2	17.3	25.3	17.3	10.0
0.1–4.9 (low)	14.6	37.0	43.0	38.8	29.5
5–14.9 (medium)	4.8	36.3	27.7	36.4	44.3
15+ (high)	0.4	9.4	4.1	7.5	16.2
**Disease prevalence (%)** ^**I**^					
Hypertension	34.1	55.2	50.6	55.4	59.4
Eye conditions*	46.8	48.3	50.0	48.5	46.6
Atrial Fibrillation	15.0	25.8	23.0	26.1	28.0
Cerebrovascular Disease	7.1	18.6	13.8	17.4	24.3
Diabetes	9.3	19.8	17.7	19.3	22.4
Heart Failure	8.3	19.3	16.0	19.3	22.4
Solid Neoplasms	19.7	21.6	22.0	21.9	21.0
Ischemic Heart Disease	12.2	19.4	17.9	19.5	20.8
Colitis and related	10.4	17.3	15.2	16.8	19.6
Peripheral Neuropathy	8.0	14.7	13.4	14.3	16.4
Osteoarthritis	11.6	13.7	14.5	13.2	13.4
COPD	3.6	9.7	9.2	9.8	10.2
Dementia	1.5	9.3	4.6	8.3	14.8
Other neurological disease^†^	2.3	8.6	5.6	7.4	12.3
Parkinson disease	0.6	3.1	1.7	2.8	4.6
Multiple sclerosis	0.1	0.6	0.3	0.5	0.9
**Number of chronic** **conditions** ^**‡**^ **(N)**					
0–1	31.2	12.9	15.9	13.2	9.8
2–4	39.8	30.8	34.2	31.2	27.1
5–9	25.6	43.0	39.8	42.9	46.0
10+	3.4	13.3	9.9	12.7	17.1
Any neurol. disease (%)	9.5	26.1	19.3	24.5	34.0
Any neurol. disease^§^ and dementia (%)	0.3	2.9	1.2	2.3	5.0
**Hospitalization during follow-up**					
Person-years at risk	231,696	207,910	74,536	70,580	62,794
Total number of hospitalizations	96,842	181,786	52,413	62,763	66,610
Number of hospitalizations per person (%)					
0	61.1	39.1	43.9	37.8	35.8
1	21.6	27.7	26.3	27.9	28.7
2+	17.3	33.2	29.8	34.3	35.5
Incidence per person-year	0.42	0.87	0.70	0.89	1.06
Proportion unplanned (%)	86.9	91.0	89.5	91.2	92.1

* Cataract, Glaucoma, Blindness, or other eye disease, see [18]

^†^ Chronic neurological conditions not otherwise specified, see [18]

^‡^ Sum of chronic conditions defined by [18]

^§^ Prevalence of at least one of the following neurological diseases: cerebrovascular disease, Parkinson disease, multiple sclerosis, epilepsy, or chronic neurological conditions not otherwise specified, see [18]

^I^ Differences in disease prevalence between the home care and non-home care group statistically significant (*p* < 0.001 for χ2-test) for all conditions. Differences in disease prevalence between home care groups statistically significant (*p* < 0.001) for all variables

During the two-year follow-up period, 28.9% of HC recipients die compared to 11.1% among the non-HC recipients. Almost 60% of the HC recipients are hospitalized at least once during the two years of follow-up, compared to less than 40% in the non-HC group. Two thirds of HC recipients live alone, and the percentage of those cohabiting decreases with increasing number of HC hours. The median amount of HC provided is 22 h per month. More than 70% of HC recipients receive personal care such as assistance with dressing or meals. Compared to non-HC recipients, HC recipients more often live alone, have a higher degree of frailty, and suffer from more chronic diseases.

### Morbidity

Table 1 shows the prevalence of the most common chronic diseases, multimorbidity, and frailty among HC recipients. The prevalence of all included diseases in the total study population and stratified by age, sex, and amount of HC is summarized in the supplement (Tables S2-S4). Among HC recipients, the most common diseases are hypertension (55.2%), eye conditions (48.3%), and atrial fibrillation (25.8%). Every other HC recipient has at least 5 chronic conditions. We also observe sex differences among HC recipients; men have a higher prevalence of cardiovascular and renal diseases as well as diabetes and stroke than women (Table S2).

For most diseases a higher prevalence correlates with a higher number of HC hours. The correlation between HC hours and disease prevalence is strongest for cerebrovascular disease, neurological diseases such as Parkinson and dementia with up to 3-fold higher prevalence in those with > 40 h of HC compared to those with < 10 h. However, this trend is not seen for eye conditions, cancer, and osteoarthritis.

**Fig. 1:**
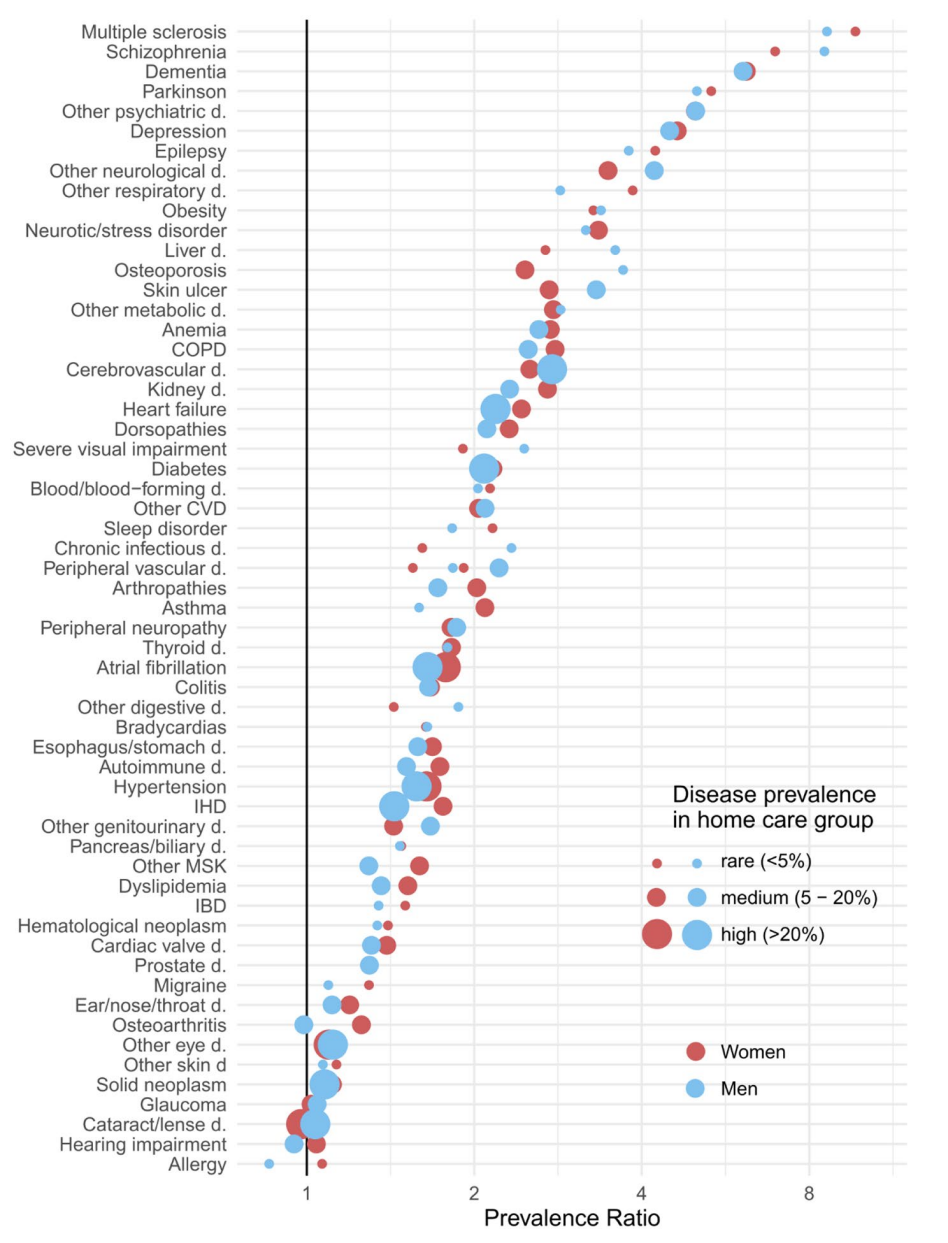
Prevalence ratios (x-axis) and prevalence (size of dots) of chronic diseases among home care recipients compared to non-home care recipients. Diseases sorted by overall prevalence in both groups combined. *COPD: Chronic obstructive pulmonary disease; CVD: Cardiovascular disease; d.: disease; IBD: Inflammatory bowel disease; IHD: Ischemic heart disease*

Figure 1 shows the prevalence ratio comparing HC recipients to non-HC recipients (indicated by the x-axis) as well as prevalence (indicated by the size of the dots) of chronic conditions in both groups. Men and women with HC have a higher prevalence of most diseases compared to non-HC recipients. The figure shows, however, that for some diseases the relative difference is particularly large such as for neurological diseases, psychiatric diseases and dementia with a 2- and 8-fold higher prevalence among HC recipients than non-HC recipients. Moreover, HC recipients have a higher prevalence of chronic kidney disease, heart failure, chronic obstructive pulmonary disease (COPD), hypertension, and obesity in both women and men. The differences in disease prevalence between HC recipients and non-HC recipients are larger in younger age groups (Tables S3-4). A few diseases such as cancer, eye conditions, and hearing impairment have a similar prevalence for the two groups.

### Risk of hospitalization

In total 60.9% of the HC recipients are hospitalized at least once during the two-year follow-up compared to 38.9% of non-HC recipients (Table 1). HC recipients are twice as often hospitalized repeatedly (> 1 times) during the follow-up compared to non-HC recipients (33.2 vs. 17.3%). Hospitalization rates increase with the number of provided HC hours (Table 1).

**Fig. 2:**
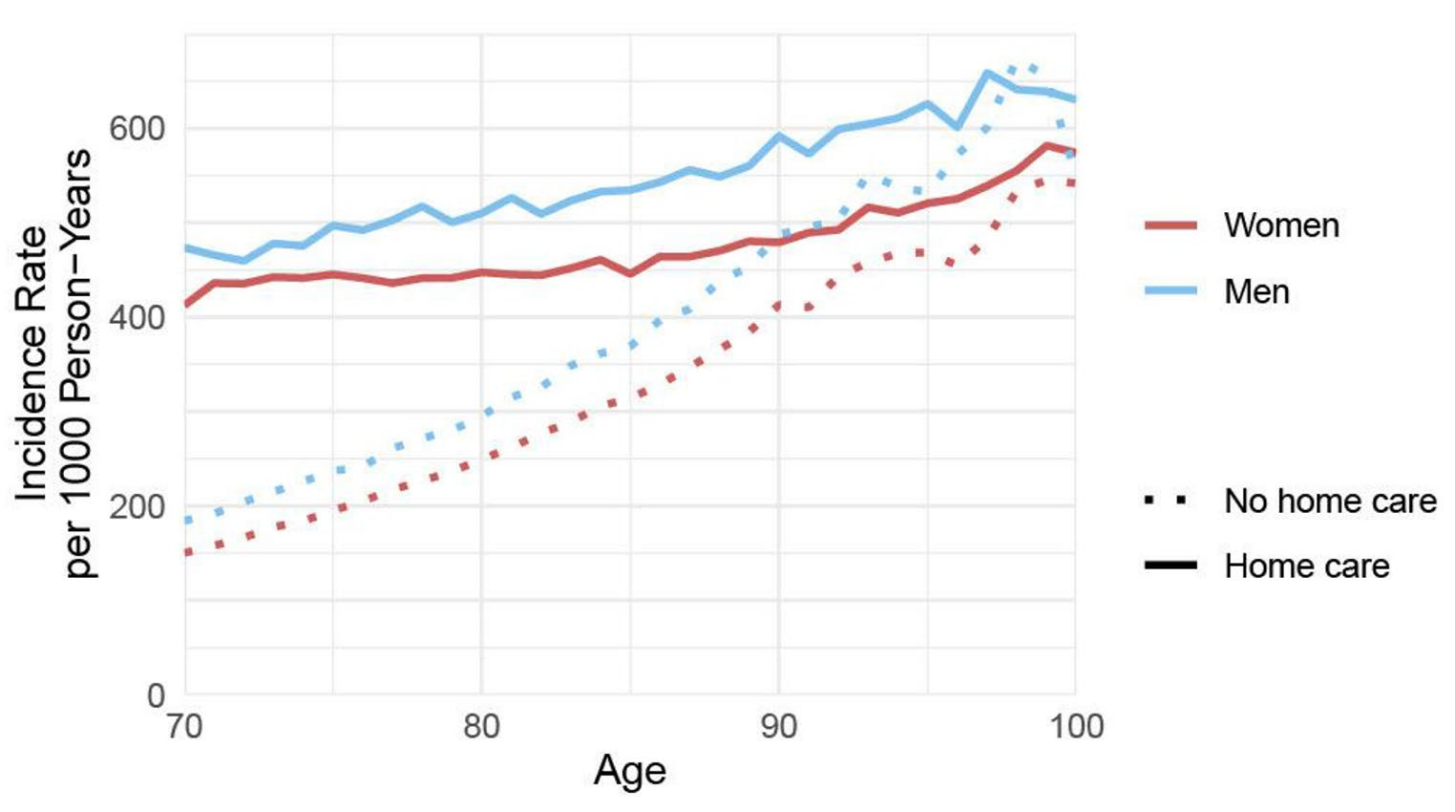
Hospitalization rate in home care recipients compared to non-home care recipients, 2019–2020

Figure 2 shows incidence rates of hospitalizations per 100 person-years among HC recipients and non-HC recipients. Across all ages, men have higher hospitalization rates than women both among HC recipients and non-HC recipients. Differences between hospitalization rates are larger at younger ages but remain up to 95 years of age (Fig. 2). At age 70, for instance, HC recipients have hospitalization rates 3 times higher than non-HC recipients.

### Reasons for hospitalizations

Table 2 shows common reasons for hospitalizations together with incidence rates and IRR comparing HC recipients with non-HC recipients. More than one third (37.4%) of hospitalizations among HC recipients are related to injuries (including hip fracture), infections (including respiratory and urinary) and heart failure (Table 2).

**Table 2:** Common reasons for hospitalization among home care recipients (*N* = 136,113). Incidence rates calculated as number of hospitalizations divided by number of person-years lived during 2019–2020 among home care recipients. Incidence rate ratios calculated as incidence rate in the home care population divided by incidence rate among non-home care recipients

	Number of hospitalizations* N = 181,786	IR per 100 person-years in home care population			IRR compared to non-home care recipients
		Total	Women	Men	
Injury other than hip fracture *S chapter excl. S72; T07, T14*	14,843	7.14	7.44	6.30	2.32
Respiratory infection *J06, J12-42, J69*	12,450	5.99	4.66	8.72	2.92
Heart failure I*50, J81*	11,475	5.51	5.09	6.77	2.07
Urinary tract infection *N10, N11, N12, N30, N32, N39*	10,333	4.97	4.05	6.86	3.10
Other infections and sepsis *A, R50*	7825	3.76	3.09	5.14	2.74
Hip Fracture *S72*	7713	3.71	3.94	3.24	1.90
COPD *J43-J47*	6321	3.04	3.11	2.89	5.06
Neoplasms *C chapter*	6193	2.98	2.61	3.07	1.21
Ischemic Stroke *I63-I69*	4655	2.24	2.07	2.59	1.62
IHD *I20-I25*	3785	1.82	1.60	2.27	1.20
COVID-19 *U07*	3341	1.61	1.42	1.98	3.12
Dizziness *R55, R42*	3284	1.58	1.51	0.84	1.43
Confusion, delirium *F05-F19, G30*	3133	1.51	1.34	1.86	4.48
Atrial fibrillation *I48*	3119	1.50	1.61	1.28	1.12
Liver, gall and pancreas diseases *K65-K91*	3072	1.48	1.33	1.77	1.56
Breathing difficulties *J96, J80, J98, R06*	2864	1.38	1.30	1.75	2.76

*main diagnoses for all hospital admissions. Admissions starting on the day of discharge for a previous admission excluded

COPD: Chronic obstructive pulmonary disease; IHD: Ischemic Heart Disease; IR: Incidence rate; IRR: Incidence rate ratio

Compared to non-HC recipients, HC recipients have 3- to 5-fold higher incidence rates of hospitalizations for COPD, confusion, infections, and breathing difficulties. Men with HC have higher rates of hospitalization for respiratory, urinary, and other infections compared to women with HC, whereas women are hospitalized more often for atrial fibrillation and hip fracture. Aside from COVID-19, our sensitivity analyses reveal no substantial differences in hospitalization patterns when restricting the follow-up to the year 2019 (Table S5). However, admissions for respiratory infections other than COVID-19 were slightly more common during 2019. Results including all main diagnoses recorded during hospitalizations yield results similar to our main analyses.

## Discussion

### Summary

In this population-wide study we show that older HC recipients are a highly multi-diseased group. Every second HC recipient has five or more chronic diseases and neurological conditions and dementia are heavily overrepresented compared to an age- and sex- matched community-dwelling population without HC. The most common diseases are cardiovascular and eye conditions, but the variety of prevalent diseases paints a diverse picture of morbidity among HC recipients.

Our findings also show that HC recipients are hospitalized 1.6 times as often as non-HC recipients and four times as likely for COPD and confusion. The rate of hospitalizations due to ischemic heart disease or neoplasms, however, was only slightly elevated among HC recipients. Among HC recipients, most common reasons for hospitalizations were injuries, infections, and heart failure.

### Comparison with existing literature

To our knowledge, this is the first population-wide study examining a comprehensive range of chronic diseases in a nationwide population of HC recipients. Former studies in HC populations focused on subgroups such as newly registered HC recipients [15] or people residing in a single municipality [20], analyzed only a small number of conditions [21], or used solely healthcare data from inpatient records. These methodological differences likely explain some notable differences in disease prevalence but also prevalence of hospitalization between our and previous studies. For instance, we observe a lower prevalence of dementia in our study (9%) compared to two studies from Finland (29%) [15] and Canada (22%) [8]. Both studies used resident assessments of dementia conducted during HC visits [22] while we used data from administrative registers which have limited sensitivity in capturing dementia. It is also possible that there are differences in who is granted HC between countries [12]. Furthermore, we find a lower percentage hospitalized at least once compared to Finnish HC recipients (60.9% of Swedish HC recipients during 2 years vs. 43% of Finnish HC recipients during one year) [15]. By contrast, the reasons for hospitalizations in our study are comparable to those described among Finnish [15], Norwegian [20], and North American [23] HC recipients.

### Implications for research and practice

We show that HC recipients are a heterogeneous group with varying morbidity patterns and hours of HC received. Cerebrovascular and neurological diseases are considerably more common among HC recipients who have a high amount of HC hours compared to those having a lower amount. Neurological diseases often cause long-term functional decline [24] which is a probable explanation for their over-representation among those with high amounts of HC and among HC recipients in general. The diversity in morbidity patterns suggests that one approach likely does not fit all and that person-centered care approaches should be considered to meet HC recipients’ care needs [14]. 

Moreover, we show that HC recipients are more often hospitalized than non-HC recipients. While on the one hand, being cared for in one’s home could potentially serve as a protection from ending up in the emergency room, the higher levels of multimorbidity and frailty in the HC population on the other hand, are risk factors for hospitalization. In frail people, even an otherwise innocent symptom or disease (e.g. lower urinary tract infection) can quickly set off a cascade of events leading to failure of several organs, severe disease symptoms and need of emergency admission. The hospitalization rate is particularly elevated among HC recipients compared to non-HC recipients within the age group 70 to 85. In this age group, people with HC are a highly selected population in comparatively poor health. Consequently, receiving HC seems to be a more powerful indicator of differences in hospitalization risk than age itself.

One third of hospitalizations among HC recipients results from injuries, infections, and heart failure. Both infections and heart failure are included in the list of 19 ambulatory-care sensitive conditions, wherein effective management and timely intervention hold the potential to prevent hospital admission [25]. For example, the majority of urinary infections manifest in the lower urinary tract could be addressed within PC if detected early. Additionally, measures such as home adjustments, walking aids, and adequate caregiver support may contribute to the prevention of fall injuries [26]. These findings contribute to the identification of vulnerabilities within the HC population, empowering clinicians to strategically target preventative measures.

Notably, aside from COVID-19 infection itself, reasons for hospitalization do not differ between 2019 and 2020 except for decreased numbers of hospitalizations for respiratory infections during 2020, perhaps resulting from older persons’ reluctance to visit emergency departments due to fear of COVID-19 [27].

The care of older community-dwelling adults with functional decline is a considerable challenge for PC professionals [28, 29] who feel overwhelmed by the complexity of health problems in frail older people [30]. Our findings point towards several potential points of action to improve the care of HC recipients. First, both disease-specific care by outpatient specialists and person-centered PC have been suggested to meet care needs of people with multimorbidity [14]. In HC recipients, the collaboration between PC and specialist caregivers, such as neurologists and rehabilitative medicine, should be promoted. Second, HC and health care are disintegrated in many countries [31] including Sweden which implies that PC staff, for instance, may not even be aware that a patient receives HC (Box 1). Contrary, qualitative studies have reported that HC staff lack opportunities to report their observations to medical professionals [32, 33]. Since the integration of PC and HC is indispensable as both partners have important knowledge to share, integration and communication should be promoted. Future research should examine if an intervention incorporating elements of the suggested points of action including continued and timely care may reduce hospitalizations in community-dwelling older people with HC.

### Strengths and limitations

Our study has several strengths including its register-based nationwide design and large size which allowed us to include all HC recipients, stratify by amount of HC provided, and to conduct comparisons with an age- and sex- matched community-dwelling cohort without HC. The Swedish National Patient Register provides high-quality data on all inpatient and specialized outpatient care in the country [16]. However, our paper also has limitations. Register data may not always convey the underlying cause for hospitalization adequately and causes may, in some cases, be multifactorial. PC stands for a substantial part of medical care for older people. Ideally, PC data should have been included but such data are not available for the entire population in Sweden. Many diseases such as depression, hypertension, hypothyroidism, or heart failure [34] may mainly be treated in PC and the prevalence of some chronic diseases is hence likely underestimated in our study. However, in Sweden, persons with severe neurological disease, e.g., Parkinson disease usually meet neurologists in regular intervals [35]. One should also note that only 13% of HC recipients do not have multimorbidity which suggests that we were able to capture a substantial part of diseases among the old. Although coverage of the Social Service Register is high in 2019 [17], a few municipalities have not reported data consistently. Therefore, we may have identified the vast majority but not the entirety of HC recipients in Sweden.

## Conclusion

Nine % of the 70 + population in Sweden rely on formal HC and the vast majority of them suffer from multiple chronic diseases. Every second HC recipient has five or more chronic diseases. The variety of prevalent diseases paints a diverse picture of morbidity among HC recipients. Almost two thirds of HC recipients are admitted to hospital at least once during the two-year follow up and the most common reasons are falls and decompensated heart failure. Compared to non-HC recipients, HC recipients more often live alone, have a higher degree of frailty, suffer from more chronic diseases and especially neurological disease, and are hospitalised almost twice as often. Hospitalisations for COPD and confusion were four times more common than in non-HC recipients. Future work should explore if and how interventions in PC and HC could lower the hospitalization rate in this vulnerable group.

### Electronic supplementary material

Below is the link to the electronic supplementary material.

Supplementary Material 1

## Data Availability

Data were provided by the Swedish National Board of Health and Welfare and Statistics Sweden. Restrictions apply to the availability of these data, which are thus not publicly accessible. Pseudonymized data are, however, available from the authors upon reasonable request and with permission of the regional ethics board in Stockholm. Statistical code is available upon request from the corresponding author.

## Declarations

### Authors’ information

KSM and CA are general practitioners and work clinically with frail older community-dwelling people. A significant part of their patients receives home care. The clinical interpretation of the findings roots mainly in their clinical experience.

## Abbreviations

COPD: Chronic obstructive pulmonary disease
HC: Home care
ICD: International Classification of Diseases
PC: Primary care
