# Supplementary material for: Profiling chronic diseases and hospitalizations in older home care recipients: a nationwide cohort study in Sweden
Source: BMC Geriatr. 2024 Apr 3;24:312. doi: 10.1186/s12877-024-04796-7 (PMC10993481; doi:10.1186/s12877-024-04796-7)
Supplement: Supplementary file 1 — Supplementary Material 1 [file 12877_2024_4796_MOESM1_ESM.docx]

**Supplementary Materials**

*Table S1: Data Sources*

|  | **Variables** | **Data Source** | **Reference** |
| --- | --- | --- | --- |
| **Elder care** | - care home residence - granted home care claims | Social Service Register (SSR) | *Meyer AC et al. Scandinavian Journal of Public Health. 2022 Nov;50(7):946-58.* |
|  |  |  |  |
| **Socioeconomic and demographic** | - Date of birth - Sex - place of residence - birth country - dates of international migration | Total Population Register | *Ludvigsson JF et al. European journal of epidemiology. 2016 Feb;31:125-36.* |
|  | Date of death | Cause of Death Register | *Brooke HL et al. European journal of epidemiology. 2017 Sep;32:765-73.* |
|  | Cohabitation | Dwelling Register | *Not available* |
|  | Education, income | Longitudinal Integration Database of Health and Labour Market Studies (LISA) (and Population Censuses) | *Ludvigsson JF et al. European journal of epidemiology. 2019 Apr 15;34:423-37.* |
|  |  |  |  |
| **Health** | Dates of and diagnoses made in inpatient and specialized outpatient care | National Patient Register (NPR) | *Ludvigsson JF et al. BMC public health. 2011 Dec;11(1):1-6.* |

*Table S2: Prevalence of chronic diseases among home care patients and age-and-sex-matched older adults without care in total, stratified by sex, and amount of home care.*

|  | | **No care** | **Home care** |  |  | **Home care** | | | | | |
| --- | --- | --- | --- | --- | --- | --- | --- | --- | --- | --- | --- |
| Chronic disease^a^ | | **Total**  **N=136,113** | **Total**  **N=136,113** |  |  | **Low amount**  **N=43,538** | **Medium amount**  **N=45,705** | **High amount**  **N=46,870** |  | **Women**  **N=89,701** | **Men**  **N=46,412** |
| Allergy | | 1.05% | 1.05% |  |  | 1.22% | 1.07% | 0.88% |  | 1.14% | 0.87% |
| Anemia | | 5.29% | 14.24% |  |  | 10.79% | 13.99% | 17.69% |  | 14.01% | 14.68% |
| Asthma | | 2.65% | 5.18% |  |  | 4.73% | 5.13% | 5.65% |  | 6.10% | 3.40% |
| Atrial fibrillation | | 14.95% | 25.75% |  |  | 22.98% | 26.05% | 28.03% |  | 23.14% | 30.80% |
| Autoimmune d. | | 3.98% | 6.65% |  |  | 6.01% | 6.72% | 7.17% |  | 7.47% | 5.06% |
| Severe visual impairment | | 0.98% | 2.04% |  |  | 1.53% | 1.96% | 2.58% |  | 1.94% | 2.21% |
| Blood/blood-forming d. | | 1.28% | 2.68% |  |  | 2.25% | 2.79% | 2.99% |  | 2.33% | 3.36% |
| Bradycardias | | 1.68% | 2.76% |  |  | 2.54% | 2.90% | 2.81% |  | 1.96% | 4.29% |
| Cardiac valve d. | | 4.84% | 6.57% |  |  | 6.45% | 6.80% | 6.46% |  | 6.25% | 7.20% |
| Cataract/lense d. | | 34.24% | 34.08% |  |  | 36.11% | 34.65% | 31.62% |  | 35.17% | 31.96% |
| Cerebrovascular d. | | 7.12% | 18.63% |  |  | 13.83% | 17.39% | 24.29% |  | 16.08% | 23.56% |
| Chromosomal abnormormalities | | 0.00% | 0.02% |  |  | 0.01% | 0.02% | 0.01% |  | 0.01% | 0.03% |
| Chronic infectious d. | | 0.08% | 0.15% |  |  | 0.12% | 0.15% | 0.19% |  | 0.13% | 0.18% |
| Kidney disease | | 3.06% | 7.66% |  |  | 6.34% | 7.52% | 9.02% |  | 5.97% | 10.94% |
| Liver disease | | 0.29% | 0.89% |  |  | 0.87% | 0.84% | 0.95% |  | 0.76% | 1.14% |
| Pancreas/biliary d. | | 2.37% | 3.50% |  |  | 3.19% | 3.49% | 3.79% |  | 3.33% | 3.83% |
| Skin ulcer | | 2.19% | 6.40% |  |  | 4.30% | 5.80% | 8.94% |  | 6.10% | 6.98% |
| Colitis | | 10.38% | 17.28% |  |  | 15.23% | 16.84% | 19.62% |  | 17.66% | 16.55% |
| COPD | | 3.63% | 9.73% |  |  | 9.22% | 9.76% | 10.16% |  | 9.37% | 10.42% |
| Hearing impairment | | 6.21% | 6.26% |  |  | 6.42% | 6.16% | 6.22% |  | 6.33% | 6.13% |
| Dementia | | 1.53% | 9.37% |  |  | 4.60% | 8.32% | 14.82% |  | 8.67% | 10.71% |
| Depression | | 1.35% | 6.19% |  |  | 7.21% | 8.92% | 10.84% |  | 9.90% | 7.37% |
| Diabetes | | 9.33% | 19.84% |  |  | 17.71% | 19.25% | 22.39% |  | 17.27% | 24.81% |
| Dorsophathies | | 4.62% | 10.40% |  |  | 8.76% | 9.89% | 12.41% |  | 11.51% | 8.25% |
| Dyslipidemia | | 6.46% | 9.37% |  |  | 9.38% | 9.41% | 9.34% |  | 8.45% | 11.16% |
| Ear/nose/throat d. | | 6.89% | 8.03% |  |  | 8.44% | 7.99% | 7.68% |  | 8.19% | 7.72% |
| Epilepsy | | 0.66% | 2.64% |  |  | 1.76% | 2.51% | 3.59% |  | 2.19% | 3.51% |
| Esophagus/stomach d. | | 4.66% | 7.66% |  |  | 6.95% | 7.80% | 8.19% |  | 7.44% | 8.08% |
| Glaucoma | | 13.62% | 13.98% |  |  | 14.25% | 13.90% | 13.80% |  | 14.58% | 12.80% |
| Heart failure | | 8.30% | 19.33% |  |  | 15.98% | 19.33% | 22.44% |  | 17.74% | 22.40% |
| Hematological neoplasm | | 1.24% | 1.70% |  |  | 1.66% | 1.75% | 1.68% |  | 1.44% | 2.19% |
| Hypertension | | 34.13% | 55.22% |  |  | 50.59% | 55.41% | 59.35% |  | 55.34% | 55.00% |
| Arthropathies | | 3.86% | 7.33% |  |  | 6.57% | 7.31% | 8.05% |  | 7.05% | 7.86% |
| IBD | | 0.70% | 1.01% |  |  | 0.99% | 1.07% | 0.98% |  | 1.00% | 1.04% |
| IHD | | 12.16% | 19.44% |  |  | 17.94% | 19.54% | 20.75% |  | 16.38% | 25.36% |
| Migraine | | 0.69% | 0.86% |  |  | 0.87% | 0.82% | 0.89% |  | 1.05% | 0.50% |
| Multiple sclerosis | | 0.06% | 0.55% |  |  | 0.34% | 0.45% | 0.86% |  | 0.62% | 0.44% |
| Neurotic/stress d. | | 1.57% | 5.17% |  |  | 4.27% | 5.18% | 5.98% |  | 5.90% | 3.74% |
| Obesity | | 0.73% | 2.40% |  |  | 2.10% | 2.40% | 2.68% |  | 2.38% | 2.44% |
| Osteoarthritis | | 11.63% | 13.67% |  |  | 14.45% | 13.24% | 13.36% |  | 15.59% | 9.97% |
| Osteoporosis | | 3.47% | 8.87% |  |  | 7.09% | 8.58% | 10.80% |  | 12.04% | 2.72% |
| Other CVD | | 5.56% | 11.46% |  |  | 9.91% | 11.35% | 13.01% |  | 9.70% | 14.85% |
| Other digestive d. | | 0.81% | 1.23% |  |  | 1.22% | 1.24% | 1.24% |  | 1.36% | 0.98% |
| Other eye d. | | 27.27% | 30.02% |  |  | 30.77% | 29.96% | 29.39% |  | 31.79% | 26.62% |
| Other genitourinary d. | | 6.80% | 10.23% |  |  | 8.64% | 9.61% | 12.30% |  | 10.22% | 10.25% |
| Other metabolic d. | | 1.70% | 4.75% |  |  | 3.41% | 4.46% | 6.27% |  | 5.10% | 4.07% |
| Other MSK | | 4.47% | 6.59% |  |  | 6.00% | 5.98% | 7.74% |  | 6.54% | 6.70% |
| Other neurological d. | | 2.29% | 8.63% |  |  | 5.62% | 7.74% | 12.31% |  | 7.22% | 11.37% |
| Other psychiatric d. | | 1.88% | 9.41% |  |  | 7.22% | 9.02% | 11.83% |  | 7.84% | 12.44% |
| Other respiratory d. | | 0.94% | 3.20% |  |  | 2.58% | 3.13% | 3.83% |  | 2.94% | 3.70% |
| Other skin d. | | 1.44% | 1.59% |  |  | 1.62% | 1.57% | 1.59% |  | 1.61% | 1.57% |
| Parkinson | | 0.59% | 3.07% |  |  | 1.72% | 2.76% | 4.61% |  | 2.25% | 4.65% |
| Peripheral neuropathy | | 8.04% | 14.72% |  |  | 13.37% | 14.33% | 16.36% |  | 15.54% | 13.14% |
| Peripheral vascular d. | | 2.01% | 4.10% |  |  | 3.59% | 3.85% | 4.81% |  | 3.51% | 5.23% |
| Prostate d. | | 4.43% | 5.74% |  |  | 5.43% | 5.70% | 6.06% |  | 0.00% | 16.82% |
| Schizophrenia | | 0.15% | 1.13% |  |  | 0.87% | 1.17% | 1.32% |  | 1.18% | 1.03% |
| Sleep disorder | | 1.31% | 2.59% |  |  | 2.39% | 2.68% | 2.70% |  | 2.06% | 3.62% |
| Solid neoplasm | | 19.73% | 21.63% |  |  | 22.00% | 21.90% | 21.02% |  | 18.43% | 27.81% |
| Thyroid d. | | 5.16% | 9.38% |  |  | 8.13% | 9.45% | 10.47% |  | 12.17% | 3.98% |
| Venous/lymphatic d. | | 1.88% | 3.05% |  |  | 2.63% | 3.03% | 3.47% |  | 3.26% | 2.65% |
|  | ^a^ defined according to Calderón-Larrañaga A, Vetrano DL, Onder G, et al. Assessing and Measuring Chronic Multimorbidity in the Older Population: A Proposal for Its Operationalization. J Gerontol A Biol Sci Med Sci 2017;72(10):1417-1423.  COPD: Chronic obstructive pulmonary disease, CVD: Cardiovascular disease; d.: disease; IBD: Inflammatory bowel disease; IHD: Ischemic heart disease; MSK: Musculoskeletal condition | | | | | | | | | | |

*Table S3: Prevalence of chronic diseases among home care patients and age-and-sex-matched older adults without care stratified by age among women.*

|  | **Women** | | | | | | | | |
| --- | --- | --- | --- | --- | --- | --- | --- | --- | --- |
|  | **70-79 years** | |  | **80-89 years** | |  | | **90+ years** | |
| Chronic disease^a^ | **No care**  **N=20,863** | **Home care**  **N=20,863** |  | **No care**  **N=44,775** | **Home care**  **N=44,775** |  |  | **No care**  **N=24,063** | **Home care**  **N=24,063** |
| Allergy | 1.47% | 1.45% |  | 1.07% | 1.18% |  |  | 0.73% | 0.80% |
| Anemia | 3.20% | 15.12% |  | 5.24% | 14.05% |  |  | 6.55% | 12.98% |
| Asthma | 3.13% | 7.26% |  | 3.07% | 6.25% |  |  | 2.46% | 4.83% |
| Atrial fibrillation | 7.20% | 16.44% |  | 13.89% | 24.45% |  |  | 16.45% | 26.51% |
| Autoimmune d. | 4.58% | 8.36% |  | 4.66% | 7.96% |  |  | 3.41% | 5.78% |
| Severe visual impairment | 0.49% | 1.51% |  | 0.96% | 1.88% |  |  | 1.58% | 2.45% |
| Blood/blood-forming d. | 0.96% | 2.73% |  | 1.13% | 2.42% |  |  | 1.14% | 1.84% |
| Bradycardias | 0.58% | 1.32% |  | 1.19% | 1.92% |  |  | 1.75% | 2.59% |
| Cardiac valve d. | 2.61% | 4.52% |  | 5.07% | 7.00% |  |  | 5.05% | 6.35% |
| Cataract/lense d. | 30.58% | 33.48% |  | 40.36% | 38.31% |  |  | 32.58% | 30.81% |
| Cerebrovascular d. | 4.50% | 17.97% |  | 6.77% | 16.27% |  |  | 7.31% | 14.08% |
| Chromosomal abnormormalities | 0.00% | 0.04% |  | 0.00% | 0.00% |  |  | 0.00% | 0.00% |
| Chronic infectious d. | 0.04% | 0.19% |  | 0.11% | 0.14% |  |  | 0.07% | 0.08% |
| Kidney disease | 1.39% | 5.87% |  | 2.27% | 5.93% |  |  | 2.78% | 6.13% |
| Liver disease | 0.51% | 1.87% |  | 0.27% | 0.57% |  |  | 0.10% | 0.15% |
| Pancreas/biliary d. | 2.26% | 3.66% |  | 2.20% | 3.28% |  |  | 2.36% | 3.15% |
| Skin ulcer | 1.61% | 7.29% |  | 2.12% | 5.73% |  |  | 2.99% | 5.75% |
| Colitis | 10.17% | 19.80% |  | 11.30% | 17.98% |  |  | 9.59% | 15.21% |
| COPD | 3.89% | 15.85% |  | 3.68% | 9.01% |  |  | 2.29% | 4.42% |
| Hearing impairment | 5.10% | 5.07% |  | 6.36% | 6.43% |  |  | 6.41% | 7.24% |
| Dementia | 0.89% | 10.10% |  | 1.47% | 9.53% |  |  | 1.74% | 5.85% |
| Depression | 1.80% | 11.41% |  | 1.44% | 6.07% |  |  | 1.05% | 3.33% |
| Diabetes | 7.88% | 22.76% |  | 8.93% | 18.10% |  |  | 6.35% | 10.96% |
| Dorsophathies | 4.93% | 12.87% |  | 5.42% | 12.08% |  |  | 4.21% | 9.26% |
| Dyslipidemia | 5.89% | 11.08% |  | 6.48% | 9.17% |  |  | 3.57% | 4.83% |
| Ear/nose/throat d. | 7.15% | 8.94% |  | 7.33% | 8.61% |  |  | 5.72% | 6.74% |
| Epilepsy | 0.57% | 3.96% |  | 0.53% | 2.02% |  |  | 0.46% | 0.98% |
| Esophagus/stomach d. | 4.94% | 9.63% |  | 4.79% | 7.64% |  |  | 3.30% | 5.18% |
| Glaucoma | 10.03% | 9.20% |  | 14.95% | 15.10% |  |  | 16.90% | 18.28% |
| Heart failure | 3.18% | 13.95% |  | 7.35% | 17.66% |  |  | 10.76% | 21.18% |
| Hematological neoplasm | 1.02% | 1.71% |  | 1.16% | 1.54% |  |  | 0.81% | 1.04% |
| Hypertension | 25.09% | 51.46% |  | 35.98% | 56.87% |  |  | 37.00% | 55.85% |
| Arthropathies | 3.77% | 8.62% |  | 3.75% | 7.45% |  |  | 2.75% | 4.96% |
| IBD | 0.96% | 1.64% |  | 0.68% | 0.96% |  |  | 0.37% | 0.51% |
| IHD | 5.49% | 13.04% |  | 9.71% | 16.54% |  |  | 11.92% | 18.98% |
| Migraine | 1.02% | 1.43% |  | 0.86% | 1.01% |  |  | 0.54% | 0.79% |
| Multiple sclerosis | 0.19% | 1.90% |  | 0.04% | 0.32% |  |  | 0.01% | 0.04% |
| Neurotic/stress d. | 2.27% | 10.02% |  | 1.78% | 5.38% |  |  | 1.31% | 3.32% |
| Obesity | 1.31% | 5.60% |  | 0.77% | 1.93% |  |  | 0.13% | 0.42% |
| Osteoarthritis | 14.42% | 17.53% |  | 13.85% | 16.90% |  |  | 8.06% | 11.46% |
| Osteoporosis | 3.44% | 11.71% |  | 5.55% | 12.67% |  |  | 4.89% | 11.17% |
| Other CVD | 3.15% | 9.61% |  | 4.99% | 9.93% |  |  | 5.75% | 9.38% |
| Other digestive d. | 1.01% | 1.96% |  | 1.09% | 1.41% |  |  | 0.64% | 0.75% |
| Other eye d. | 20.39% | 25.04% |  | 30.19% | 32.24% |  |  | 34.34% | 36.78% |
| Other genitourinary d. | 8.08% | 11.48% |  | 7.38% | 10.56% |  |  | 5.84% | 8.47% |
| Other metabolic d. | 1.92% | 6.65% |  | 1.88% | 4.83% |  |  | 1.70% | 4.25% |
| Other MSK | 5.87% | 9.43% |  | 4.12% | 6.38% |  |  | 2.53% | 4.32% |
| Other neurological d. | 2.22% | 11.51% |  | 2.19% | 6.85% |  |  | 1.73% | 4.18% |
| Other psychiatric d. | 2.19% | 14.34% |  | 1.53% | 6.97% |  |  | 1.11% | 3.82% |
| Other respiratory d. | 0.85% | 5.15% |  | 0.84% | 2.72% |  |  | 0.55% | 1.43% |
| Other skin d. | 1.39% | 1.60% |  | 1.42% | 1.59% |  |  | 1.44% | 1.65% |
| Parkinson | 0.55% | 4.07% |  | 0.49% | 2.29% |  |  | 0.18% | 0.59% |
| Peripheral neuropathy | 8.19% | 17.54% |  | 9.20% | 15.96% |  |  | 7.65% | 13.02% |
| Peripheral vascular d. | 1.37% | 4.38% |  | 2.01% | 3.43% |  |  | 1.92% | 2.90% |
| Prostate d. | 0.00% | 0.00% |  | 0.00% | 0.00% |  |  | 0.00% | 0.00% |
| Schizophrenia | 0.29% | 3.29% |  | 0.15% | 0.68% |  |  | 0.09% | 0.26% |
| Sleep disorder | 1.54% | 3.78% |  | 0.94% | 1.92% |  |  | 0.47% | 0.84% |
| Solid neoplasm | 14.74% | 18.08% |  | 16.81% | 18.62% |  |  | 17.71% | 18.39% |
| Thyroid d. | 5.05% | 11.59% |  | 7.16% | 12.61% |  |  | 7.22% | 11.85% |
| Venous/lymphatic d. | 2.34% | 3.52% |  | 2.21% | 3.38% |  |  | 1.70% | 2.81% |
| ^a^ defined according to Calderón-Larrañaga A, Vetrano DL, Onder G, et al. Assessing and Measuring Chronic Multimorbidity in the Older Population: A Proposal for Its Operationalization. J Gerontol A Biol Sci Med Sci 2017;72(10):1417-1423.  COPD: Chronic obstructive pulmonary disease, CVD: Cardiovascular disease; d.: disease; IBD: Inflammatory bowel disease; IHD: Ischemic heart disease; MSK: Musculoskeletal condition | | | | | | | | | |

*Table S4: Prevalence of chronic diseases among home care patients and age-and-sex-matched older adults without care stratified by age among men.*

|  | **Men** | | | | | | | | |
| --- | --- | --- | --- | --- | --- | --- | --- | --- | --- |
|  | **70-79 years** | |  | **80-89 years** | |  | | **90+ years** | |
| Chronic disease^a^ | **No care**  **N=15,266** | **Home care**  **N=15,266** |  | **No care**  **N=22,028** | **Home care**  **N=22,028** |  |  | **No care**  **N=9118** | **Home care**  **N=9118** |
| Allergy | 1.18% | 0.95% |  | 1.07% | 0.85% |  |  | 0.61% | 0.79% |
| Anemia | 3.67% | 14.90% |  | 6.38% | 14.46% |  |  | 7.04% | 14.86% |
| Asthma | 1.88% | 3.00% |  | 2.40% | 3.77% |  |  | 1.91% | 3.15% |
| Atrial fibrillation | 12.56% | 24.55% |  | 21.19% | 33.30% |  |  | 22.86% | 35.19% |
| Autoimmune d. | 3.28% | 5.37% |  | 3.76% | 5.16% |  |  | 2.48% | 4.31% |
| Severe visual impairment | 0.37% | 1.78% |  | 1.06% | 2.15% |  |  | 1.41% | 3.10% |
| Blood/blood-forming d. | 1.42% | 3.22% |  | 1.82% | 3.49% |  |  | 1.65% | 3.29% |
| Bradycardias | 1.11% | 2.46% |  | 3.05% | 4.70% |  |  | 4.04% | 6.37% |
| Cardiac valve d. | 3.90% | 5.85% |  | 6.66% | 8.16% |  |  | 5.43% | 7.14% |
| Cataract/lense d. | 21.68% | 26.29% |  | 36.05% | 35.29% |  |  | 33.66% | 33.38% |
| Cerebrovascular d. | 6.33% | 26.35% |  | 9.46% | 23.37% |  |  | 10.02% | 19.37% |
| Chromosomal abnormormalities | 0.01% | 0.05% |  | 0.00% | 0.02% |  |  | 0.00% | 0.00% |
| Chronic infectious d. | 0.09% | 0.25% |  | 0.10% | 0.17% |  |  | 0.04% | 0.09% |
| Kidney disease | 2.82% | 9.96% |  | 5.37% | 11.19% |  |  | 6.35% | 11.94% |
| Liver disease | 0.53% | 2.65% |  | 0.26% | 0.51% |  |  | 0.10% | 0.10% |
| Pancreas/biliary d. | 2.04% | 3.59% |  | 2.79% | 3.97% |  |  | 3.10% | 3.88% |
| Skin ulcer | 1.41% | 8.67% |  | 2.28% | 6.30% |  |  | 2.86% | 5.81% |
| Colitis | 8.84% | 17.08% |  | 10.79% | 16.85% |  |  | 10.01% | 14.95% |
| COPD | 3.68% | 12.37% |  | 4.82% | 10.68% |  |  | 3.40% | 6.50% |
| Hearing impairment | 5.57% | 4.61% |  | 6.85% | 6.45% |  |  | 7.02% | 7.90% |
| Dementia | 1.08% | 11.25% |  | 2.12% | 12.00% |  |  | 2.03% | 6.67% |
| Depression | 1.52% | 8.36% |  | 1.14% | 4.78% |  |  | 0.87% | 2.18% |
| Diabetes | 12.14% | 30.92% |  | 13.18% | 24.86% |  |  | 8.42% | 14.44% |
| Dorsophathies | 3.68% | 8.68% |  | 4.43% | 8.74% |  |  | 3.06% | 6.33% |
| Dyslipidemia | 8.70% | 13.23% |  | 9.00% | 11.56% |  |  | 5.43% | 6.72% |
| Ear/nose/throat d. | 6.73% | 8.03% |  | 7.30% | 7.78% |  |  | 6.50% | 7.06% |
| Epilepsy | 0.85% | 5.44% |  | 0.97% | 2.98% |  |  | 0.95% | 1.58% |
| Esophagus/stomach d. | 4.83% | 9.71% |  | 5.60% | 7.99% |  |  | 4.35% | 5.58% |
| Glaucoma | 8.31% | 8.55% |  | 13.15% | 14.03% |  |  | 16.75% | 16.94% |
| Heart failure | 5.90% | 18.05% |  | 11.44% | 23.78% |  |  | 14.69% | 26.35% |
| Hematological neoplasm | 1.40% | 2.36% |  | 1.86% | 2.22% |  |  | 1.48% | 1.80% |
| Hypertension | 29.63% | 55.00% |  | 38.06% | 56.31% |  |  | 36.15% | 51.81% |
| Arthropathies | 4.36% | 8.14% |  | 4.96% | 8.28% |  |  | 4.00% | 6.39% |
| IBD | 1.00% | 1.40% |  | 0.77% | 1.00% |  |  | 0.39% | 0.54% |
| IHD | 13.02% | 21.37% |  | 19.85% | 26.84% |  |  | 20.10% | 28.46% |
| Migraine | 0.43% | 0.44% |  | 0.50% | 0.58% |  |  | 0.42% | 0.43% |
| Multiple sclerosis | 0.09% | 0.96% |  | 0.04% | 0.24% |  |  | 0.02% | 0.04% |
| Neurotic/stress d. | 1.19% | 5.74% |  | 1.20% | 3.07% |  |  | 1.11% | 2.00% |
| Obesity | 1.26% | 4.97% |  | 0.59% | 1.58% |  |  | 0.13% | 0.26% |
| Osteoarthritis | 11.20% | 9.71% |  | 10.71% | 10.87% |  |  | 6.71% | 8.21% |
| Osteoporosis | 0.45% | 2.76% |  | 0.90% | 2.82% |  |  | 0.80% | 2.40% |
| Other CVD | 5.43% | 15.03% |  | 8.20% | 15.39% |  |  | 7.23% | 13.25% |
| Other digestive d. | 0.35% | 1.19% |  | 0.65% | 0.95% |  |  | 0.53% | 0.72% |
| Other eye d. | 16.25% | 20.75% |  | 26.25% | 28.01% |  |  | 30.98% | 33.06% |
| Other genitourinary d. | 5.96% | 10.91% |  | 6.58% | 10.59% |  |  | 5.42% | 8.34% |
| Other metabolic d. | 1.59% | 5.14% |  | 1.43% | 3.78% |  |  | 1.13% | 2.99% |
| Other MSK | 6.66% | 8.78% |  | 4.92% | 6.29% |  |  | 3.33% | 4.19% |
| Other neurological d. | 2.57% | 16.23% |  | 2.88% | 10.44% |  |  | 2.47% | 5.49% |
| Other psychiatric d. | 3.49% | 21.26% |  | 2.37% | 9.68% |  |  | 1.11% | 4.35% |
| Other respiratory d. | 1.04% | 4.87% |  | 1.57% | 3.51% |  |  | 1.06% | 2.18% |
| Other skin d. | 1.24% | 1.02% |  | 1.46% | 1.70% |  |  | 1.84% | 2.15% |
| Parkinson | 1.10% | 6.94% |  | 0.99% | 4.40% |  |  | 0.48% | 1.39% |
| Peripheral neuropathy | 6.47% | 13.85% |  | 7.69% | 13.41% |  |  | 6.54% | 11.31% |
| Peripheral vascular d. | 1.81% | 6.56% |  | 2.77% | 4.91% |  |  | 2.28% | 3.81% |
| Prostate d. | 11.24% | 13.48% |  | 14.27% | 18.39% |  |  | 12.78% | 18.63% |
| Schizophrenia | 0.18% | 2.34% |  | 0.10% | 0.49% |  |  | 0.07% | 0.13% |
| Sleep disorder | 2.95% | 5.63% |  | 1.81% | 3.23% |  |  | 0.78% | 1.22% |
| Solid neoplasm | 20.45% | 23.18% |  | 27.96% | 29.69% |  |  | 29.78% | 31.05% |
| Thyroid d. | 1.62% | 3.43% |  | 2.44% | 4.17% |  |  | 2.73% | 4.46% |
| Venous/lymphatic d. | 1.38% | 2.94% |  | 1.43% | 2.55% |  |  | 1.62% | 2.41% |
| ^a^ defined according to Calderón-Larrañaga A, Vetrano DL, Onder G, et al. Assessing and Measuring Chronic Multimorbidity in the Older Population: A Proposal for Its Operationalization. J Gerontol A Biol Sci Med Sci 2017;72(10):1417-1423.  COPD: Chronic obstructive pulmonary disease, CVD: Cardiovascular disease; d.: disease; IBD: Inflammatory bowel disease; IHD: Ischemic heart disease; MSK: Musculoskeletal condition | | | | | | | | | |

*Table S5: Most common causes of hospitalization among home care patients (N=136,113) during the entire follow-up and during 2019 only.*

|  | **Entire period (2019-2020)** | | **2019 only** | |
| --- | --- | --- | --- | --- |
|  | **Number of hospitalizations**  **N=181,786** | **Proportion of total (%)** | **Number of hospitalizations**  **N=106,745** | **Proportion of total (%)** |
| Injury other than hip fracture  *S chapter excl. S72; T07, T14* | 14,843 | 8.2 | 8289 | 7.8 |
| Respiratory infection  *J06, J12-42, J69* | 12,450 | 6.8 | 8138 | 7.6 |
| Heart failure  I*50, J81* | 11,475 | 6.3 | 7099 | 6.7 |
| Urinary tract infection  *N10, N11, N12, N30, N32, N39* | 10,333 | 5.7 | 6075 | 5.7 |
| Other infections and sepsis  *A, R50* | 7825 | 4.3 | 4500 | 4.2 |
| Hip Fracture  *S72* | 7713 | 4.2 | 4168 | 3.9 |
| COPD  *J43-J47* | 6321 | 3.5 | 3985 | 3.7 |
| Neoplasms  *C chapter* | 6193 | 3.4 | 4086 | 3.8 |
| Ischemic Stroke  *I63-I69* | 4655 | 2.6 | 2726 | 2.6 |
| IHD  *I20-I25* | 3785 | 2.1 | 2245 | 2.1 |
| Covid-19  *U07* | 3341 | 1.8 | N/A | N/A |
| Dizziness  *R55, R42* | 3284 | 1.8 | 1956 | 1.8 |
| Confusion, delirium  *F05-F19, G30* | 3133 | 1.7 | 1938 | 1.8 |
| Atrial fibrillation  *I48* | 3119 | 1.7 | 1821 | 1.7 |
| Liver, gall and pancreas diseases  *K65-K91* | 3072 | 1.7 | 1809 | 1.7 |
| Breathing difficulties  *J96, J80, J98, R06* | 2864 | 1.6 | 1539 | 1.4 |
